# Supplementary material for: Competition between electron pairing and phase coherence in superconducting interfaces
Source: Nat Commun. 2018 Jan 29;9:407. doi: 10.1038/s41467-018-02907-8 (PMC5789063; doi:10.1038/s41467-018-02907-8)
Supplement: Supplementary file 1 — Supplementary Information [file 41467_2018_2907_MOESM1_ESM.pdf]

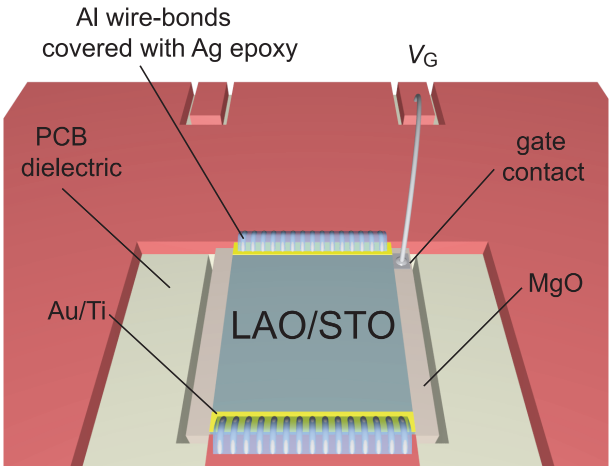

Supplementary Figure 1: **$LaAlO_3/SrTiO_3$ sample in the CPW transmission line.** Close up view on the $LaAlO_3/SrTiO_3$ sample inserted between the central strip and the ground of a CPW transmission line. The sample is glued on a MgO substrate and contacted through multiple Al wire-bonds covered by silver epoxy to ensure negligible impedance contacts. A tiny metallic contact on the MgO substrate allows the gate to be connected to an external contact pad through an Al wire-bond.

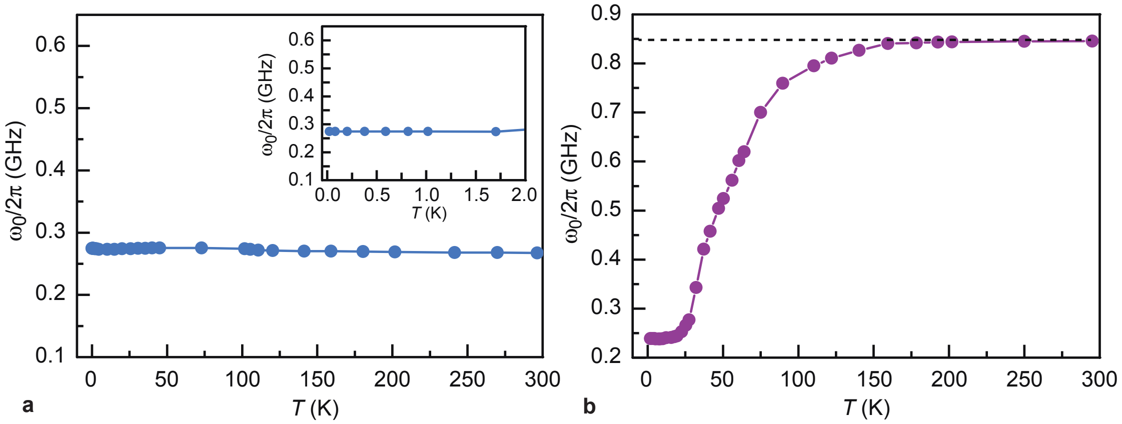

Supplementary Figure 2: **Resonant RLC circuits with SMD devices** a) Resonance frequency of a RLC circuit made with SMD devices (L = 14.5 nH and C = 24 pF) as a function of temperature. The nominal resonance frequency is $\omega_0/2\pi$ = 269.8 MHz and the measured one at T $\simeq$ 0 K is $\omega_0/2\pi$ = 274.7 MHz. Inset) Resonance frequency in the low temperature range. b) Resonance frequency of the $LaAlO_3/SrTiO_3$ sample circuit as a function of temperature. The shift is due to the increase of the $SrTiO_3$ dielectric constant entering into C_{STO} when the temperature is lowered. Below 10 K quantum fluctuations lead to a saturation of the dielectric constant.

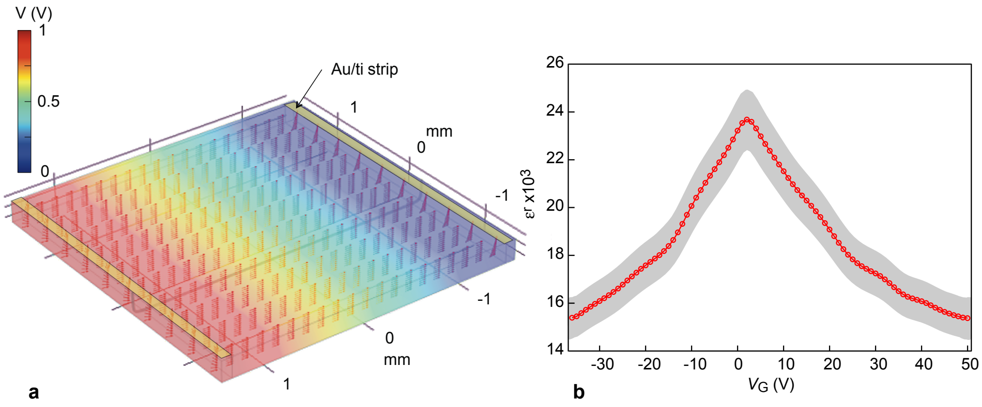

Supplementary Figure 3: **Numerical simulation of $SrTiO_3$ dielectric constant.** a) Finite element method numerical simulation of the potential distribution (color scale) in the $\mathrm{SrTiO_3}$ substrate for a 1 V potential applied on one Au/Ti strip while the other one is at the ground. Arrows indicate the direction of the electric field. b) Dielectric constant computed from the value of $(C_{\mathrm{STO}} - C_{\mathrm{para}})$ determined experimentally. The grey outline indicates the error margin due to the uncertainty of the exact geometry of the $\mathrm{SrTiO_3}$ subtrate.

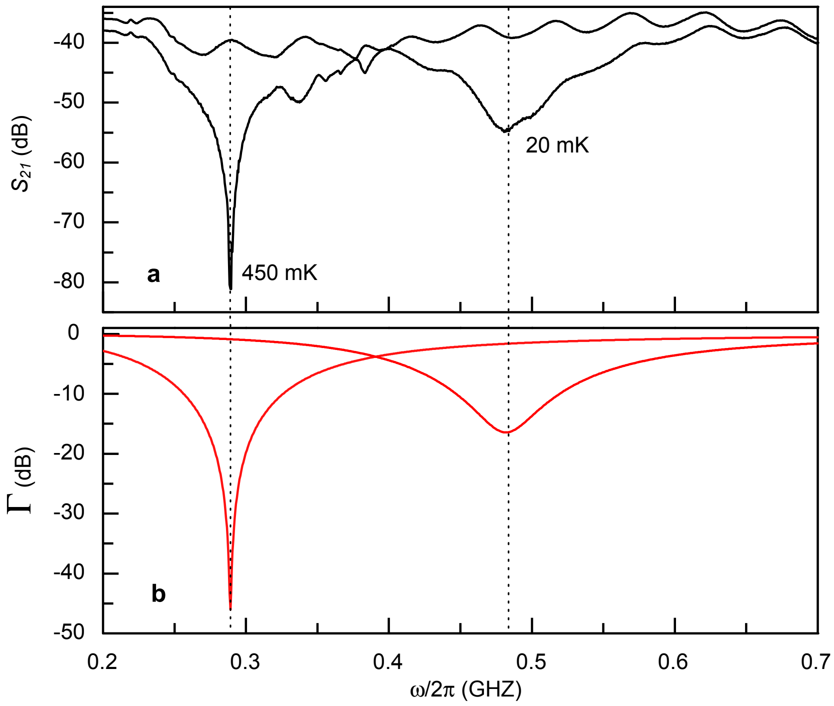

Supplementary Figure 4: **Comparison between raw data and calibrated data.** a) Magnitude of the transmission coefficient S_{21} measured with the VNA at T = 450 mK and T = 20 mK for V_{G}= 50 V. The resonance frequency is clearly visible on both curves. b) Reflection coefficient Γ of the sample circuit extracted from S_{21} after calibration (see Methods section).

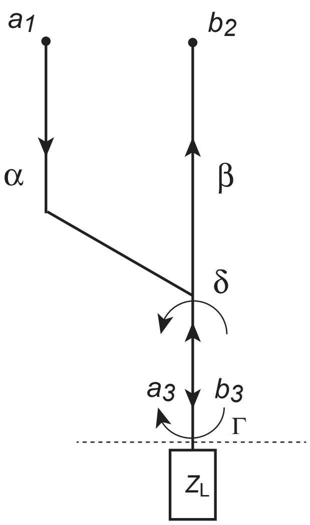

Supplementary Figure 5: **Modeling of the microwave set-up using the scattering matrix formalism**. a_i and b_i denote the complex amplitude of the incoming and outgoing waves at point i. We assume that the VNA connected to port 1 and 2 is perfectly matched to the 50Ω coaxial cables of the set-up, which implies that no signal is reflected at port 1 and port 2. $\alpha(\omega)$, $\beta(\omega)$, $\gamma(\omega)$ and $\delta(\omega)$ are complex coefficients representing the transmission and reflection coefficients in the various points of the circuit, which satisfy the relations $a_3 = \delta b_3 + \alpha a_1$ and $b_2 = \gamma a_1 + \beta b_3$. The transmission coefficient between port 1 and port 2 can be expressed as $S_{21}(\omega) = b_2(\omega)/a_1(\omega)$ and the reflection coefficient of the sample circuit can be expressed as $\Gamma(\omega) = b_3(\omega)/a_3(\omega)$.

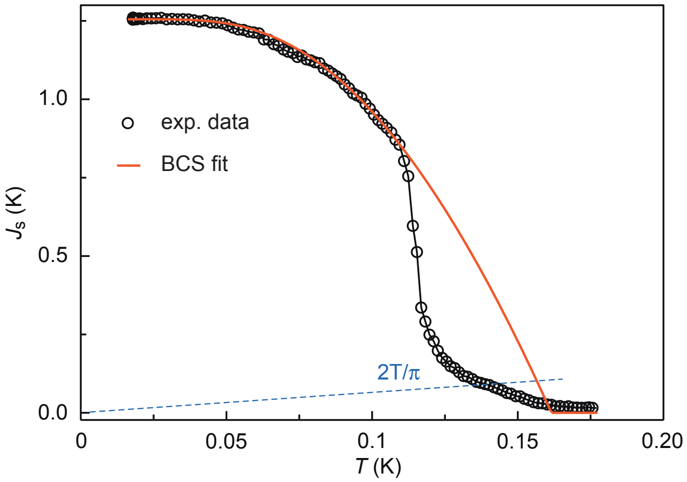

Supplementary Figure 6: **Temperature dependence of the superfluid stiffness.** BCS fit of the temperature dependence of $J_{\rm s}^{\rm exp}$ at $V_{\rm G} = 34$ V close to optimal doping. The gap energy extracted from the fit is $\Delta(0) = 24$ μeV. The blue dashed line indicate the universal BKT line $\frac{2T}{\pi}$.

Supplementary Note 1: $LaAlO_3/SrTiO_3$ heterostructure and sample circuit

In this work, 8 uc-thick $LaAlO_3$ epitaxial layers were grown on 3×3 mm^2 TiO_2-terminated (001) $SrTiO_3$ single crystals by Pulsed Laser Deposition (see Methods section for details on the growth). A weakly conducting metallic back-gate has bee deposited on the backside of the 200 μm thick substrate. Its very high resistance (∼100 kΩ) avoids any short-cut of the microwave signal by the gate. A close-up view of the sample environment is shown on Supplementary Figure 1. Two 100 μm wide and 3 mm long Ti/Au strips are deposited at the edges of the sample to obtain good electrical contacts. The sample is glued with GE Varnish on a 1 mm thick MgO substrate to isolate the gate from the electric ground of the cryostat. A section of the CPW transmission line is removed and the $LaAlO_3/SrTiO_3$ sample is inserted between the central line and the ground of the CPW. Multiple wire-bonds to the Ti/Au stripes covered by silver epoxy ensure a negligible impedance contacts ($<$ 0.1 Ω) between the 2-DEG and the CPW line both at DC and RF frequency. A wire-bond allows connecting the gate with a tiny metallic contact on the MgO substrate.
The sample circuit is designed into a laminate consisting of a h =1.3 mm thick ceramic-filled PTFE composite having a dielectric constant of 10.5 at low temperature covered by a 50 μm thick copper layer. The CPW transmission line has a s = 3 mm wide central line separated from the ground by a gap W = 1.1 mm. Its characteristic impedance is Z_0 = 50 $\pm 1\Omega$ [1]. The CPW line is soldered to a SMA microwave connector, which is in turn connected to a semi-rigid coaxial cable.

Surface Mounted microwave Devices (SMD) inductor L_1 and resistor R_1 are connected in parallel to the $LaAlO_3/SrTiO_3$ heterostructure to form a RLC resonant circuit. The capacitor of the resonant circuit is formed by the intrinsic capacitance of the $SrTiO_3$ in parallel of the 2-DEG. Two protective capacitors C_p = 2 μF are added in series with inductor L_1 and resistor R_1 to prevent the dc current from flowing through L_1 and R_1. However, because of their large capacitance they do not influence the resonance frequency. SMD microwave inductors and resistors have been extensively tested at low temperature to ensure their reliability. Supplementary Figure 2a shows the temperature dependence of the resonance frequency $\omega_0 = \frac{1}{\sqrt{L_1 C_1}}$ of a RLC circuit where the $LaAlO_3/SrTiO_3$ sample has been replaced by a SMD capacitor C_1. The experimental value of ω_0 corresponds to the one calculated from the nominal values of L_1 and C_1 within less than 5% of error. It varies by less than 3% over the entire temperature range 20 mK - 300 K and by less than 0.1% below 1 K. More over, this weak variation of ω_0 with temperature is likely due to the capacitor C_1 which is replaced by the sample in the real experiment.

The value of L_1, which is determined by the geometrical inductance of the inductor, is not expected to vary with temperature. Four points DC measurements also confirm that the resistance R_1 doesn't change significantly with temperature ($< 0.5\%$). In any case, R_1 doesn't enter into the determination of the resonance frequency and consequently in the determination of J_s.

Supplementary Note 2: Accuracy on the determination of J_s

The total capacitance of the circuit is extracted from the resonance frequency in the normal state. Supplementary Figure 2b shows the temperature dependence of ω_0 of the sample circuit in the temperature range 0.4 - 300 K which illustrates clearly the quantum paraelectric nature of $SrTiO_3$. Above 10 K, the resonance frequency increases with temperature because of the reduction of the dielectric constant. The saturation of ω_0 above 200 K is due to the parasitic capacitance of the circuit $C_{para} \simeq$ 3.5 pF. Below 10 K, quantum fluctuations lead to a saturation of the dielectric constant and ω_0 becomes temperature independent. This is in particular true in the temperature range of interest ($<$ 500 mK) as seen in Fig. 3a of the manuscript. A low temperature, the total capacitance of the circuit is mainly dominated by C_{STO} ($\simeq$ 45 pF for V_G = 0 V). Although it is the total capacitance of circuit that is measured in the normal state and plotted in Fig. 2c, we refer to it as C_{STO} for sake of clarity ($C_{STO}+C_{para} \simeq C_{STO}$).

The determination of the superfluid stiffness J_s^{exp} relies on the measurement of the kinetic inductance L_k, which is extracted from the resonance frequency ω_0 in the superconducting state. This latter depends only on the total inductance and capacitance of the sample circuit. The total resistance of the circuit is not involved in the determination of J_s but it must be sufficiently close to Z_0=50 Ω to generate a visible absorption dip. The accuracy of the method can be estimated from the uncertainty in the value of the total inductance of the circuit in the normal state. In practice, we assume that by design, L_1 dominates the total inductance of the circuit. This SMD component has a nominal value of 10 nH with a tolerance of 5%. In addition we neglected in first approximation the geometrical inductance of the 2-DEG which can be estimated with the following relation [2]

$$L_G = 0.2l[\ln\frac{2l}{w+t} + 0.5 + 0.02235\frac{w+t}{l}] \quad [\text{nH}]$$

where l is the length of the 2-DEG expressed in mm (3mm), w is its width (3 mm) and t its thickness (~10 nm). As $t \ll w$, L_G is essentially independent of t and we obtain $L_G \simeq 0.85$ nH. The error margin

in the determination of J_s^{exp}, taking into account the 5% tolerance on L_1 and the contribution of the geometrical inductance, is indicated by a grey outline in Figure 4a of the main text. It is lower than 15 % in the entire phase diagram.

Supplementary Note 3: Temperature dependence of J_s^{exp}

In the overdoped region where the zero-temperature stiffness coincides with the BCS prediction, the gap energy can be extracted from a fit of the temperature dependence of $J_s^{exp}(T)$ [3]. As seen in Supplementary Figure 6, a good agreement is obtained between experimental data at $V_G = 34$ V and the BCS model in the low temperature part of the curve ($T < T_c/2$) for $\Delta(0) = 24$ μeV. This value is very close from $\Delta_s^{exp} = 22.2$ μeV that was obtained from the conversion of $J_s^{exp}(0)$ into a gap energy through Eq. (2). On the other hand, at higher temperature, $J_s^{exp}(T)$ deviates from the BCS prediction in a Berezinskii-Kosterlitz-Thouless like jump as expected in the presence of vortex fluctuations. However, the jump occurs before the intersection with the universal line $\frac{2T}{\pi}$ and it is smeared out. A quantitative analysis of this issue, which must include the contribution of spatial inhomogeneities [4], will require more further theoretical developments.

Supplementary References

[1] Gupta, K. C. Microstrip Lines and Slotlines, second Edition, Artech House, Boston (1996).

[2] Terman, F. E. Radio Engineers Handbook, McGraw-Hill, New York, (1945).

[3] Ganguly, R., Chaudhuri, D., Raychaudhuri, P. Benfatto, L. Slowing down of vortex motion at the Berezinskii-Kosterlitz-Thouless transition in ultrathin NbN films. *Phys. Rev B* **91**, 054514 (2015).

[4] Maccari, I., Benfatto, L. Castellani, C. Broadening of the Berezinskii-Kosterlitz-Thouless transition by correlated disorder. *Phys. Rev B* **96**, 060508(R) (2017).
